# A Supervised, Online, Home-Based Eccentric Resistance Exercise Program for Patients With Metabolic Dysfunction-Associated Steatotic Liver Disease

**DOI:** 10.14740/gr2131

**Published:** 2026-04-27

**Authors:** Kedar S. Deshpande, Kazunori Nosaka, Aus Molan, Isaac S. Raj, John K. Olynyk, Oyekoya T. Ayonrinde

**Affiliations:** a Exercise Medicine Research Institute, School of Medical and Health Sciences, Edith Cowan University, Perth, Australia; b Pathwest Laboratory Medicine, Perth, Australia; c School of Allied Health Sciences, Charles Darwin University, Darwin, Australia; d Curtin Medical Research Institute, Curtin University, Perth, Australia; e Department of Gastroenterology and Hepatology, Fiona Stanley Hospital, Perth, Australia; f Medical School, University of Western Australia, Perth, Australia

**Keywords:** Controlled attenuation parameter, Liver stiffness, Liver enzyme, Lipid profile, Physical function test, Exercise adherence

## Abstract

**Background:**

Metabolic dysfunction-associated steatotic liver disease (MASLD) is the most common non-communicable chronic liver disease worldwide, increasing long-term risk of cirrhosis, type 2 diabetes mellitus, and cardiovascular diseases. Although reducing liver steatosis by increasing physical activity can lower these risks, patients with MASLD often struggle to exercise regularly. We examined the effects of a supervised, home-based, online bodyweight eccentric exercise program on liver and metabolic markers, and physical fitness in patients with MASLD.

**Methods:**

Sixteen adults with MASLD (24–91 years) were assigned to an exercise group (n = 9) or a control group receiving lifestyle counselling (n = 7). The exercise group participants performed an online progressive bodyweight eccentric exercise program targeting trunk, leg, and arm muscles for 5 to 25 min per session, 5 days a week, for 8 weeks. Outcome measures included severity of hepatic steatosis (controlled attenuation parameter (CAP)), anthropometric measures (waist and hip circumferences), liver enzymes, and physical function tests, which were assessed at baseline and after the 8-week period.

**Results:**

All participants in the exercise group completed 40 exercise sessions, and showed reductions in CAP (−13.2±13.8%, P = 0.03), waist circumference (−4.4±13.5%, P = 0.007), hip circumference (−2.6±11.1%, P = 0.03), and serum gamma-glutamyl transferase (−23.9±30.0%, P = 0.03), which were not evident in the control group. Physical fitness of the exercise group participants improved, including the sit-to-stand (24.3±33.2%, P = 0.03), 3-m timed up and go (−15.6±16.4%, P = 0.01), 2-min step test (37.5±20.5%, P = 0.008), and single-leg balance (25.1±37.6%, P = 0.04).

**Conclusion:**

The exercise program was effective in reducing hepatic steatosis and increase physical fitness in patients with MASLD, highlighting the efficacy of the home-based eccentric exercise program in enhancing liver and metabolic health.

## Introduction

Metabolic dysfunction-associated steatotic liver disease (MASLD), formerly known as non-alcoholic fatty liver disease (NAFLD) [1], is the most common non-communicable chronic liver disease, affecting approximately 30% of the adult population worldwide [2]. The hallmark of MASLD is the accumulation of triglycerides (TG) in at least 5% of hepatocytes, along with at least one metabolic dysfunction feature, excluding secondary causes such as excessive alcohol intake. MASLD encompasses a range of liver pathologies, from simple steatosis to metabolic dysfunction-associated steatohepatitis (MASH), progressing through varying severities of liver fibrosis to cirrhosis or hepatocellular carcinoma [3]. MASLD is one of the most common causes of chronic liver disease, but it is often underappreciated as a cause of cirrhosis [4].

The global burden of MASLD is substantial, with an estimated 38% of adults and 7–14% of children and adolescents currently affected worldwide, and adult prevalence projected to exceed 55% by 2040, contributing to a rising incidence of advanced liver disease and MASLD-related deaths [5, 6]. Among individuals with type 2 diabetes mellitus (T2DM), MASLD prevalence is estimated to be between 55% and 70% [7]. MASLD is associated with a two-fold increased risk of developing T2DM and cardiovascular disease [8, 9]. However, reversal of hepatic steatosis is associated with a reduced likelihood of developing these [10, 11].

Lifestyle modifications, including dietary changes and regular exercise, remain the cornerstone of MASLD treatment [12]. Both aerobic and resistance exercises have demonstrated benefits for liver enzymes, liver fat, and metabolic risk factors [13, 14]. Despite increasing evidence supporting these benefits, the optimal type, duration, intensity, and frequency of exercise for MASLD management remain unclear. Furthermore, implementing effective exercise programs in this population is challenging. Most individuals with MASLD fail to exercise regularly, citing time constraints, fatigue, pain, chronic health issues, lack of motivation, lack of enjoyment, and lack of education as the most commonly reported barriers [15, 16]. Thus, developing an exercise program that is acceptable to people with MASLD, is effective, and addresses these barriers, is essential to improving adherence and long-term sustainability.

While resistance exercises are effective, they often require specialized equipment and access to a gymnasium, which may limit accessibility and scalability. Eccentric exercise, which involves lengthening the muscle under tension, such as sitting to a chair slowly and descending stairs, has garnered attention due to its lower metabolic demand and reduced fatigue, potentially making it more suitable for individuals with MASLD [17, 18]. Compared to concentric training, eccentric exercise has been shown to produce greater increases in strength and muscle mass and to improve insulin sensitivity and lipid metabolism [19, 20].

Despite these advantages, only one study has investigated the effects of eccentric training on individuals with MASLD [21]. This study combined eccentric exercise with vitamin D supplementation in overweight women with MASLD and found significant reductions in liver enzymes alanine aminotransferase (ALT), aspartate aminotransferase (AST), and gamma-glutamyl transferase (GGT) and improvements in lipid profile. Therefore, the effects of eccentric training alone, particularly when delivered in a supervised, online, home-based format, appear to be an attractive intervention. This mode of delivery may help overcome common exercise barriers such as time constraints and travel limitations. Previous studies have demonstrated the benefits of resistance training in individuals with MASLD. For example, Takahashi et al [22] showed that simple resistance exercises, such as push-ups and squats, improved metabolic markers in this population. However, the intervention was delivered in-person and did not focus on eccentric contractions in the study. More broadly, a recent systematic review and meta-analysis by Zafar et al [23] reported that eHealth interventions, including mobile apps, telehealth, and web-based coaching, can improve liver enzymes and body composition in individuals with MASLD. While these findings support the feasibility of remote interventions, none of the included studies evaluated a fully supervised, real-time, bodyweight eccentric-only program tailored for this population. Given the physiological advantages and lower fatigue profile of eccentric exercise, its delivery in a digitally supervised, home-based format may offer a novel and accessible solution for overcoming common barriers to exercise in people with MASLD.

Therefore, the present study aimed to evaluate the effects of an 8-week online bodyweight eccentric exercise program on hepatic steatosis, liver enzymes, metabolic parameters, and physical function in patients with MASLD. We hypothesized that the intervention would be feasible and well-tolerated and would improve liver-related and metabolic health markers, whereas the control group receiving lifestyle advice would not demonstrate these changes.

## Materials and Methods

### Study design and participants

The present study was a pilot and feasibility quasi-randomized controlled trial which compared an online bodyweight eccentric exercise group with a non-exercise control group of individuals diagnosed with MASLD. This study was approved by the Human Research Ethics Committee of the South Metropolitan Health Service (RGS0000005982) and Edith Cowan University (2023-04727-DESHPANDE), and conducted in accordance with the Declarations of Helsinki and Istanbul. The trial was retrospectively registered with the Australian New Zealand Clinical Trials Registry (ANZCTR; Trial ID: ACTRN12626000100392).

We recruited adults (≥ 18 years) diagnosed with MASLD attending the hepatology outpatient clinic of a tertiary Australian hospital, identified during routine appointments based on hepatic steatosis or elevated liver enzymes. Consecutive patients were approached to minimize selection bias and improve generalizability. Participants underwent FibroScan^®^ 502 Touch to assess hepatic steatosis severity using the controlled attenuation parameter (CAP) and liver fibrosis with transient elastography (TE), respectively, following evaluation by an experienced hepatologist. MASLD diagnosis was based on a recent multi-society consensus [24]. Since a CAP score above 275 dB/m indicates a significant accumulation of liver fat [25], individuals with a CAP score of 275 dB/m or higher were invited to participate in the present study.

Exclusion criteria included other liver diseases, such as autoimmune hepatitis, viral hepatitis or significant alcohol consumption, hepatic encephalopathy, ascites, esophageal varices, consumption of weight-loss drugs, bariatric surgery, and hepatocellular carcinoma (HCC). It was a requirement that the participants had not been doing any structured resistance and/or aerobic exercise within the last 6 months.

A priori sample size estimation was performed using G*Power (version 3.1.9.7). An effect size of 0.8 was selected to represent a large effect, based on Cohen’s conventional criteria and the expectation that this pilot study would primarily detect relatively large changes in key liver-related outcomes following the intervention. Using an alpha level of 0.05 and 80% power, the estimated minimum total sample size was 12 participants. Of 25 contacted patients, 20 consented. However, three participants were excluded resulting in 17 eligible participants. Due to the quasi-randomized design, group allocation was based on availability and preference. This approach was necessary to accommodate the practical constraints faced by the participants, such as scheduling conflicts and logistical issues, while still aiming to maintain a balance between groups. Ten participants entered the exercise group and seven the control group. One participant in the exercise group withdrew after 5 weeks due to personal reasons, leaving nine in the exercise group and seven in the control group. A per-protocol analysis was conducted on those who completed the intervention.

Among the 16 participants, there were equal numbers of men and women (n = 8 each). Table 1 summarizes baseline characteristics. The two groups were similar in age, sex, ethnicity, body mass index (BMI), steatosis grade, and fibrosis stage (P > 0.05), although more participants in the exercise group had T2DM (89% vs. 29%; P = 0.01).

**Table 1:** Baseline Characteristics of Participants in the Exercise and Control Groups

	Exercise group	Control group	P value
Age (years)	57.4 ± 15.5 (26–78)	47.4 ± 23.0 (24–91)	0.22
Gender (male/female)	5/4	3/4	0.62
Ethnicity (n, %)			0.64
Indian	1 (11%)	2 (29%)	
Asian	2 (22%)	-	
Caucasian	5 (56%)	3 (43%)	
African	-	-	
Aboriginal & TSI	1 (11%)	-	
Others	-	2 (29%)	
Body mass (kg)	89.4 ± 13.0 (70–115)	91.0 ± 32.5 (63–157)	0.63
BMI (kg/m^2^)	32.4 ± 4.5 (23.4–39.7)	32.7 ± 6.4 (23.5–41.7)	0.95
Diabetes mellitus (n, %)	8 (89%)	2 (29%)	0.01*
Dyslipidemia (n, %)	4 (44%)	4 (57%)	0.62
Steatosis grade (n, %)			0.39
S1	-	-	
S2	1 (11%)	2 (29%)	
S3	8 (89%)	5 (71%)	
Fibrosis grade (n, %)			0.05
F0	5 (56%)	7 (100%)	
F1	3 (33%)	-	
F2	1 (11%)	-	

Values are expressed as mean ± SD along with the lowest-highest values of the participants or number of participants, and its percentage in the group participants. P value indicates the result of Mann–Whitney U test to compare between groups. *Significant (P < 0.05) difference between groups. BMI: body mass index; SD: standard deviation; TSI: Torres Strait Islander.

### Exercise intervention

A home-based online bodyweight eccentric exercise program was designed and supervised by the investigator in collaboration with an experienced exercise physiologist. Spanning over 8 weeks, the program consisted of five sessions per week, each progressing from 5 to 25 min. This gradual progression accommodated varying fitness levels and helped prevent participant overload. Each session began with a 5-min dynamic warm-up and ended with a 5-min static stretching cool-down for safety and recovery.

The exercises included chair squats, heel raises, abdominal reclines, and push-ups to target major muscle groups essential for functional mobility in older adults, as supported by previous studies [22, 26]. These exercises were selected based on prior study reporting their safety and suitability for individuals with metabolic and functional limitations [27]. The program followed a structured progression: week 1 involved one set of five reps per exercise; week 2, two sets of five; weeks 3–4, one set of ten; weeks 5–6, two sets of ten; and weeks 7–8, three sets of ten. The progression was structured to ensure gradual overload while accommodating individual tolerance.

The investigator accessed the participants through Google Meet invites, which allowed for real-time supervision throughout the sessions. To ensure a strong foundation for the exercises, participants received in-person instructions for the eccentric bodyweight exercises at the beginning of the study. During sessions, the investigator demonstrated movements, corrected techniques if necessary, and provided feedback. This real-time supervision enabled the investigator to closely observe the participants, offering immediate feedback on their forms and techniques. Moreover, the online format facilitated individualized support, as the investigator was able to address participants’ specific concerns and modify exercises as needed. The real-time supervision also allowed modification of exercises according to participant capability, as important component of process evaluation.

Control group participants received general lifestyle advice per European Association for the Study of the Liver (EASL) guidelines [28] and were offered the exercise program after study completion.

### Primary and secondary outcome measures

Primary outcomes included changes in hepatic steatosis severity based on FibroScan^®^ CAP scores, liver stiffness measurement (LSM), levels of AST, ALT, and GGT, blood glucose, glycosylated hemoglobin (HbA1c), insulin, low-density lipoprotein (LDL-C), high-density lipoprotein (HDL-C), total cholesterol (TC), and TG concentration in the blood. Participants fasted for at least 12 h before venesection and blood analysis at a nationally accredited diagnostic laboratory. Insulin resistance was assessed using the homeostasis model assessment insulin resistance (HOMA-IR) index calculation: HOMA-IR = fasting insulin × fasting blood glucose/22.5.

Secondary outcomes included BMI, waist circumference (WC), hip circumference, triglyceride glucose waist index (TyG-WC), fatty liver index (FLI), fibrosis-4 index (Fib-4), and physical function tests. Waist circumference was measured at the end of normal exhalation midway between the lowest rib and the top of the iliac crest, with the participant standing and relaxed. Hip circumference was measured at the widest part of the hips and buttocks at the end of exhalation in a relaxed stance.

The Fib-4 index was calculated using age, AST, ALT, and platelet levels [29]. FLI included BMI, WC, TG, and GGT levels, while TyG-WC was calculated using TG, fasting glucose, and WC [30]. Together, these tests offer a cost-effective, validated, and reliable means of noninvasively stratifying risk, monitoring progression, and informing treatment decisions in MASLD care. All assessments were conducted at baseline and after the 8-week intervention. Blood tests, CAP, and TE were performed after overnight fasting.

### Physical function tests

The following physical function tests were performed only in the exercise group. These tests were chosen based on previous studies that assessed the effects of bodyweight eccentric exercise [23]. Physical function tests were not performed by the control group participants to avoid introducing a learning effect or unintentionally encouraging participation in physical activity, which could influence the comparability of outcomes. All tests were demonstrated to participants beforehand to ensure proper understanding of the movement patterns; however, a full familiarization trial was not conducted due to time and practical constraints. These assessments have strong test–retest reliability in adults, and therefore a single well-standardized trial was considered acceptable within the clinical context of this study.

#### 30-s sit-to-stand test

To measure lower-body strength, participants were asked to stand up and sit down from a chair as many times as possible within 30 s. We counted each full stand and there was only one trial for each participant.

#### 2-min step test

Aerobic endurance was assessed. The participants were instructed to march in place, lifting each knee to a specified height, for two full minutes. The number of times the right knee reached the target height was counted. This study was a single trial.

#### 3-m timed up and go test

Functional mobility and balance were assessed. The participants started from a sitting position, stood up, walked 3 m, turned, walked 3 m back, and sat down. The researchers recorded the time required to complete the task. A trial was conducted for each participant.

#### Hand grip strength test

Grip strength was measured using a hand dynamometer as an indicator of the overall muscle strength. The participants were instructed to squeeze the dynamometer as hard as possible with each hand. Two trials were performed for each hand and the highest average score from both hands was recorded.

#### One-leg balance test

This test measures the balance, stability, and coordination. The participants were asked to stand on one leg of their choice for a maximum of 60 s without support. The time required to maintain balance was recorded. Two trials were performed for each leg and the best results were recorded.

### Survey about the exercise program

After completing the 8-week program, participants completed a brief online questionnaire (Qualtrics) to share their opinions and experiences. The survey included questions on the program’s relevance, usefulness, enjoyment, perceived difficulty, and challenges related to the online format. Participants also selected their favorite aspects from pre-populated options. These responses provided insights into the program’s acceptability and feasibility.

### Statistical analyses

Data were analyzed using IBM SPSS Statistics for Windows (Version 29.0. Armonk, NY, USA: IBM Corp). Normality and homogeneity were assessed using the Shapiro–Wilk and Levene tests, respectively. Baseline differences between groups were analyzed using the Mann–Whitney U test. Within-group changes after 8 weeks were assessed using the paired Student’s *t*-test and Wilcoxon signed-rank test. A significance level of P < 0.05 was used. Results are presented as mean ± standard deviation (SD) and range (lowest to highest value).

## Results

### Primary and secondary outcomes

Table 2 shows changes in the outcome measures from baseline to 8 weeks later for the exercise and control groups. The exercise group showed decreases (P < 0.05) in body mass, BMI, WC, and hip circumference, but these were not found in the control group. In the exercise group, CAP scores, GGT, TyG-WC index, and FLI decreased significantly over the 8-week period (P < 0.05), but no significant changes were observed in the control group. TG, HDL, LDL, glucose, insulin, HOMA-IR, and HbA1c did not show significant changes (all P > 0.05) from baseline to 8 weeks for both exercise and control groups.

**Table 2:** Changes of Variables From Baseline to 8 Weeks Later for the Exercise (n = 9) and Control (n = 7) Groups

Variable	Group	Baseline	8-week
Body mass (kg)	Exercise	89.4 ± 13.0 (70–115)	88.5 ± 13.1* (69–114)
	Control	91.0 ± 32.5 (63–157)	91.5 ± 34.8 (62–163)
BMI (kg/m^2^)	Exercise	32.4 ± 4.5 (23.4–39.7)	32.1 ± 4.5* (23.1–38.9)
	Control	32.7 ± 6.4 (23.5–41.7)	32.8 ± 7.0 (23.1–43.3)
WC (cm)	Exercise	103.5 ± 10.2 (89–117)	99.1 ± 9.6* (85–110)
	Control	104.0 ± 15.1 (85–133)	101.5 ± 17.7 (82–135)
Hip circumference (cm)	Exercise	112.8 ± 9.1 (97–127)	109.8 ± 8.5* (92–118)
	Control	117.0 ± 16.4 (98–139)	114.7 ± 17.2* (95–139)
ALT (U/L)	Exercise	58.1 ± 36.9 (22–146)	51.0 ± 42.2 (19–153)
	Control	55.1 ± 32.0 (12–109)	49.4 ± 41.2 (19–141)
AST (U/L)	Exercise	39.6 ± 15.1 (21–64)	37.3 ± 19.5 (21–80)
	Control	39.0 ± 13.5 (19–60)	38.7 ± 19.2 (17–66)
GGT (U/L)	Exercise	62.7 ± 32.06 (20–115)	47.7 ± 22.4* (23–78)
	Control	65.5 ± 83.0 (13–252)	69.5 ± 94.4* (12–282)
Triglycerides (mmol/L)	Exercise	1.3 ± 0.4 (0.8–2.1)	1.2 ± 0.3 (0.8–1.7)
	Control	1.3 ± 0.4 (0.9–2.2)	1.5 ± 0.4 (0.9–1.9)
HDL (mmol/L)	Exercise	1.1 ± 0.1 (1–1.5)	1.2 ± 0.2 (1.1–1.5)
	Control	1.1 ± 0.1 (0.9–1.4)	1.18 ± 0.2 (1–1.5)
LDL (mmol/L)	Exercise	2.8 ± 0.9 (1.1–4.2)	2.9 ± 0.9 (1.5–4.3)
	Control	2.5 ± 0.6 (1.8–3.5)	2.6 ± 0.8 (1.6–4.2)
Glucose (mmol/L)	Exercise	7.7 ± 3.6 (5.1–17.1)	7.1 ± 3.0 (4.9–14.4)
	Control	5.2 ± 0.4 (4.7–6.1)	5.2 ± 0.5 (4.7–6.0)
Insulin (mIU/L)	Exercise	13.1 ± 4.6 (7–23)	12.2 ± 5.8 (4–20)
	Control	12.5 ± 6.7 (4–24)	9.8 ± 4.4 (4–16)
HOMA-IR	Exercise	3.8 ± 1.4 (2.1–6.7)	3.4 ± 2.2 (1.1–8.0)
	Control	3.0 ± 1.8 (0.8–6.5)	2.3 ± 1.1 (0.8–4.1)
HbA1c (%)	Exercise	6.7 ± 1.8 (5.4–11.2)	6.6 ± 1.8 (5.2–11.2)
	Control	5.7 ± 0.5 (4.7–6.2)	5.6 ± 0.3 (5.0–6.0)
CAP (dB/m)	Exercise	342 ± 28.2 (300–388)	297.1 ± 37.7* (243–341)
	Control	327.8 ± 23.7 (305–376)	292.4 ± 53.6 (243–399)
LSM (kPa)	Exercise	5.9 ± 1.6 (3.5–8.3)	5.4 ± 1.08 (4.1–7.7)
	Control	5.3 ± 1.1 (3–6.6)	5.9 ± 1.0 (4.9–7.3)
TyG-WC	Exercise	498.6 ± 63.7 (412–614)	469.3 ± 57.9* (392–561)
	Control	481.4 ± 65.6 (395–599)	479.0 ± 85.4 (399–647)
FLI	Exercise	78.7 ± 17.6 (45–95)	70.6 ± 21.9* (37–93)
	Control	75.8 ± 22.3 (33–97)	73.8 ± 23.9 (35–99)
Fib-4 index	Exercise	1.3 ± 0.7 (0.6–2.5)	1.3 ± 0.7 (0.5–2.5)
	Control	1.0 ± 0.9 (0.4–3.0)	1.19 ± 1.2 (0.3–3.9)

Values are expressed as mean ± standard deviation (SD), with ranges showing the lowest and highest values for each variable. *Significant (P < 0.05) difference from the baseline. Between-group differences are described in the Results text; table values reflect within-group changes only. ALT: alanine transaminase; AST: aspartate aminotransferase; BMI: body mass index; CAP: controlled attenuation parameter; Fib-4: fibrosis-4 index; FLI: fatty liver index; GGT: gamma-glutamyl transferase; HbA1c: glycosylated hemoglobin; HDL: high-density lipoprotein; HOMA-IR: homeostasis model assessment of insulin resistance; LDL: low-density lipoprotein; LSM: liver stiffness measurement; TG: triglycerides; TyG-WC: triglyceride-glucose waist circumference index; WC: waist circumference.

**Figure 1:**
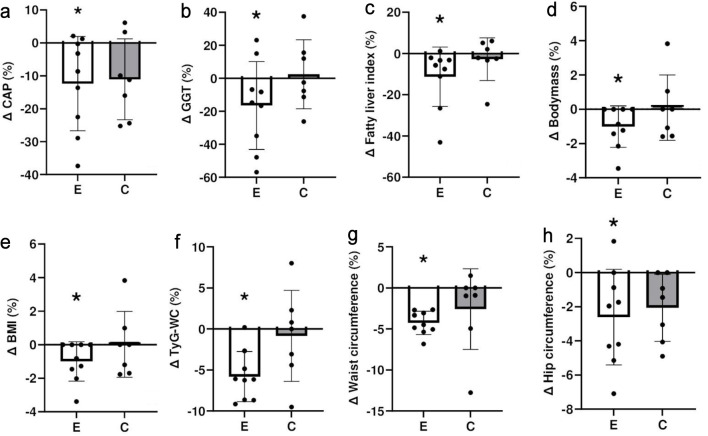
Comparison between exercise (E) and control (C) groups for percent changes in (a) controlled attenuation parameter (CAP), (b) gamma-glutamyl transferase (GGT) activity, (c) fatty liver index, (d) body mass, (e) body mass index (BMI), (f) triglyceride glucose waist index (TyG-WC), (g) waist circumference, and (h) hip circumference from baseline to 8 weeks later. *A statistically significant within-group change from baseline (P < 0.05). The figure illustrates group trends and inter-individual variability; formal between-group comparisons were not statistically significant.

Figure 1 shows the variability among participants for changes in some of the above measures from baseline to 8 weeks later. Large individual variability was evident in the percentage changes, indicating the presence of both responders and non-responders within the exercise group. Some participants experienced marked improvements, while others showed minimal or no changes, emphasizing the heterogeneous responses to the exercise intervention.

When comparing the exercise and control groups, CAP score decreased by 12% in the exercise group, GGT activity was reduced by 16%, FLI showed an 11% reduction in the exercise group, and TyG-WC demonstrated a decrease of 6% in the exercise group, but no significant changes were found in the control group. WC and hip circumference decreased by 4% and 3%, respectively, in the exercise group, while no significant changes were seen in the control group. Formal between-group comparisons did not show statistically significant differences, which is expected given the small sample size.

### Physical fitness

As shown in Table 3, all physical fitness tests showed significant improvement after the exercise intervention. A large inter-individual variability was observed for the changes, but on average, the 2-min step count showed a 38% increase, the sit-to-stand repetition had a 26% increase, the balance test time increased 90%, and the 3-m timed up and go test time decreased by 15%.

**Table 3:** Changes of Variables From Baseline to After 8-Week Training (Post-Training) for the Exercise Group

Variable	Baseline	Post-training
2-min step (n)	86.3 ± 13.0 (56–98)	118.7 ± 15.7* (79–134)
Sit-to-stand (n)	14.8 ± 2.5 (12–19)	18.4 ± 4.2* (11–25)
Single-leg balance (s)	39.7 ± 24.4 (7–60)	49.7 ± 15.2* (23–60)
Hand grip (kg)	40 ± 16.4 (19–63)	40.1 ± 16.5 (20–66)
Timed up and go (s)	6.4 ± 0.6 (5.3–7.5)	5.4 ± 0.9* (4.6–7.3)

Values are expressed as mean ± standard deviation (SD) of nine participants and their lowest and highest values in the brackets. *Significant (P < 0.05) difference from the baseline value.

### Participant feedback

Participants in the exercise group were asked whether they would continue performing the exercises after the 8-week program had concluded, and 44% of the participants responded with “probably yes” or “definitely yes,” indicating a positive outlook on continuing the exercises. Seventy-eight percent of the participants found that the program was extremely relevant, 78% reported that the exercises were somewhat enjoyable, and 22% stated that the exercises were extremely enjoyable. In terms of difficulty, 44% of the participants found the exercises were not difficult to perform, 33% reported that the exercises were somewhat difficult, and 22% found the exercises somewhat difficult to perform. Importantly, 89% of participants did not find the online format difficult to follow, and the top three reasons for liking the program were that it was supervised by a professional, home-based, and no specialized equipment was required to perform the exercises.

## Discussion

To the best of our knowledge, this was the first study to examine the effects of an online bodyweight eccentric exercise program on liver health, metabolic markers, and physical function in individuals with MASLD. The program was designed to address common barriers to exercise by offering a simple, structured, and low-fatigue solution requiring minimal equipment and time. All participants in the exercise group completed 40 sessions over 8 weeks, demonstrating high feasibility, acceptability, and adherence. The results supported the hypothesis that the exercise program would improve liver-related and metabolic health markers.

The exercise group demonstrated significant improvements in hepatic steatosis, WC, hip circumference (Table 2, Fig. 1), and physical fitness (Table 3) compared with baseline, with some notable improvements in liver enzymes and metabolic markers. These findings are consistent with previous studies supporting the effects of exercise on managing MASLD [13, 31]. Importantly, the present study uniquely employed a bodyweight, eccentric-only resistance training program delivered entirely online in real time. Eccentric exercise is metabolically less demanding and may be more suitable for individuals with MASLD who report fatigue, time constraints, or other comorbidities that limit traditional forms of exercise. The structured progression, live supervision, and real-time feedback likely contributed to the high adherence rate and effective engagement. The 100% adherence among those who completed the intervention suggests that this format may overcome common physical and psychological barriers, such as lack of motivation, pain, or lack of access to facilities. This aligns with previous research identifying these barriers as major obstacles for patients with MASLD [15]. The program’s home-based and supervised delivery model is also low-cost and scalable, offering a viable alternative to both gym-based exercise and expensive pharmacological interventions. Unlike medications that typically target advanced disease and may be financially inaccessible to many, this intervention requires no specialized equipment or infrastructure and could be delivered widely in clinical or community settings.

In the exercise group, hepatic steatosis measured by CAP decreased significantly over the 8-week intervention period. However, LSM, which reflects hepatic fibrosis rather than steatosis, did not change during the study period (Table 3). Previous studies reported mixed results regarding the effect of short-term exercise on liver stiffness. Some studies found a modest reduction in liver stiffness, whereas others suggested that longer intervention periods might be required to detect significant changes in fibrosis markers [32]. Oh et al [33] stated that only high-intensity aerobic exercise, but not moderate-intensity aerobic and resistance exercises, was effective in improving liver stiffness. The absence of change in LSM in this study may therefore be due to the short duration and the resistance-based nature of the program. Given the small sample size and quasi-randomized design, the observed reduction in CAP should be interpreted as exploratory and hypothesis-generating rather than definitive evidence of intervention efficacy.

Although glucose, insulin resistance, and lipid profiles did not change significantly, the exercise group showed meaningful reductions in WC and hip circumference (Table 3). Additionally, the present study found significant improvements in the TyG-WC and the FLI in the exercise group. These anthropometric measures and biomarkers are reliable indicators of metabolic health and are independently associated with the presence or risk of developing MASLD and other metabolic disorders [34, 35]. Reductions in central obesity, as represented by decreases in WC and hip circumference, are strongly associated with improved metabolic outcomes [36]. The reduction in WC and hip circumference aligns with previous studies that demonstrated exercise-induced improvements in body composition attributed to reductions in subcutaneous trunk fat and visceral fat [37, 38]. Although some studies have reported that resistance training could reduce visceral fat, the reduction was typically smaller compared to aerobic exercise and dietary interventions [39]. Reducing visceral fat and improving insulin sensitivity leads to better glycemic and lipid metabolism, which can help reduce liver fat accumulation and prevent its progression to fibrosis [40].

The absence of significant changes in glucose, insulin, HOMA-IR, and lipid profile may reflect the short duration of the intervention, the relatively low external load inherent to bodyweight eccentric exercise, and the high prevalence of T2DM in the exercise group. In individuals with long-standing metabolic dysfunction, improvements in glycemic or lipid measures often require longer intervention periods or higher training volumes. Therefore, the non-significant findings are not unexpected and should be interpreted within the context of the study design rather than as a lack of physiological benefit.

Improvements in FLI and TyG-WC also support the use of these indices as sensitive markers of metabolic health in MASLD populations [41–43]. Improvements in these indices suggest that the eccentric exercise program not only reduced liver fat but also improved other aspects of metabolic health. GGT is strongly associated with liver steatosis and metabolic dysfunction, and its reduction may signal a decrease in hepatic fat and oxidative stress [44]. Although changes in ALT and AST levels did not reach statistical significance, the trend towards improvement in these markers suggests the potential benefits of eccentric exercise.

There was considerable inter-individual variability in response to the intervention, as shown in Figure 1. While some participants showed marked improvements, others showed minimal or no change, despite adhering fully to the program. This heterogeneity is consistent with previous exercise trials [45] and may reflect differences in baseline health, lifestyle, motivation, or genetic predisposition. Such variability reinforces the need for personalized approaches to exercise prescription in MASLD management. Nonetheless, these improvements were not observed in the control group, suggesting that the intervention played a key role in driving these changes.

MASLD is associated with cardiovascular disease, which represents the leading cause of mortality in this population [46, 47]. Both conditions share common pathophysiological mechanisms including insulin resistance, systemic inflammation, endothelial dysfunction, and visceral adiposity. Consequently, improvements in metabolic health and central adiposity observed in the present study may have broader cardiometabolic implications beyond liver-specific outcomes. Regular exercise is known to improve endothelial function, reduce systemic inflammation, and enhance cardiometabolic health, which may partly explain the beneficial changes observed in the present study. Recent reviews have highlighted the bidirectional relationship between MASLD and cardiovascular disease and emphasize the importance of lifestyle interventions, particularly exercise, in improving both hepatic and cardiovascular risk profiles [46, 48].

The exercise group showed improvements across several physical function tests, including the 2-min step test, sit-to-stand test, single-leg balance test, and 3-m timed up and go test (Table 3). The gains indicate enhanced aerobic capacity, mobility, functional independence, muscular strength, and balance which are known benefits of eccentric training [19, 20]. Moreover, these benefits are especially important for individuals with MASLD, who often experience fatigue and reduced functional capacity. Grip strength did not improve, which is consistent with the training specificity principle. The program primarily targeted the lower body and trunk with only minimal direct loading of the forearm flexors. Additionally, several participants had T2DM, in whom reduced peripheral nerve function and handgrip responsiveness are common. Given the low absolute upper-limb training volume, minimal change in grip strength is expected and does not contradict the broader functional gains observed.

The beneficial effects observed may be partly explained by physiological mechanisms previously associated with eccentric exercise. However, because the study did not include a comparison exercise group, these improvements may not be attributed specifically to eccentric contractions and may instead reflect the general benefits of engaging in regular resistance exercise. Nonetheless, several eccentric-related mechanisms provide useful context for understanding the potential adaptations observed. Eccentric exercises are less metabolically demanding, as shown by lower oxygen consumption and heart rate for the same work in eccentric cycling than for concentric cycling [49]. This is likely to make them more tolerable for individuals with MASLD who experience fatigue [17]. Furthermore, eccentric contractions produce distinct neuromuscular responses such as enhanced central nervous activity, increased muscle-tendon stiffness, and increased muscle protein synthesis [50]. It also increases glucose transporter type 4 (GLUT4) translocation in skeletal muscle, facilitating glucose uptake independent of insulin, which may contribute to improved metabolic function [51]. Additionally, eccentric exercise stimulates the release of myokines like interleukin-6 (IL-6) and irisin, which reduce inflammation and promote lipid oxidation, potentially mitigating liver inflammation and fat storage [51]. The reductions in CAP and improvements in GGT observed in the present study may plausibly reflect improvements in insulin sensitivity, skeletal muscle glucose uptake, and myokine-mediated metabolic regulation induced by resistance exercise. While these mechanisms were not directly measured in this study, they are plausible contributors to the observed changes and warrant further investigation in future research.

Post-program feedback indicated that most participants found the exercises highly relevant, enjoyable, and easy to perform. The online format was well received, with participants highlighting the convenience of supervised, at-home sessions requiring no travel or equipment. These findings reinforce the potential of remote, supervised interventions to improve accessibility, especially in populations with low confidence, comorbidities, or logistical barriers to attending in-person programs. The ability to provide real-time feedback and modify exercises individually also supports safe implementation and high engagement.

The present study had several limitations. The small sample size and quasi-randomized design limit internal validity and generalizability. Future randomized controlled trials should incorporate formal power calculations to determine an adequate sample size capable of detecting clinically meaningful changes in hepatic and metabolic outcomes. Participants self-selected into the intervention or control group based on availability and preference, which may have introduced selection bias. Additionally, individuals who opted for the exercise group may have had greater motivation or readiness for lifestyle change, which could have influenced the observed outcomes independently of the intervention. The marked imbalance in T2DM prevalence between the groups may also have confounded metabolic outcomes, as diabetes is a major driver of MASLD progression and may influence responsiveness to lifestyle interventions. Future studies should consider stratified randomization by diabetes status and relevant medication use. However, this approach mirrors real-world conditions and improves ecological validity, as it reflects the choices patients typically make in clinical settings. The study duration was relatively short, and long-term sustainability of the observed benefits is unknown. Dietary intake was not monitored, which may have influenced metabolic markers. However, this was an intentional design choice to isolate the effects of the exercise intervention. The absence of muscle mass measurements is another limitation, given the known relationship between skeletal muscle and MASLD management via enhancing insulin sensitivity, lipid metabolism, and metabolic health [52]. Future studies should include dual-energy X-ray absorptiometry (DEXA) or bioelectrical impedance analysis (BIA) to assess changes in lean mass. Finally, while this online exercise program shows promise as a scalable intervention, it does not address barriers like internet access, digital literacy, or socioeconomic constraints. Physical function assessments were performed only in the exercise group; therefore, these improvements should be interpreted as within-group observations and not as definitive between-group intervention effects. Future studies should include these assessments in both groups.

Our findings align with recent evidence demonstrating the multi-system involvement of MASLD. A 2024 systematic review confirmed strong links between sarcopenia and MASLD progression, highlighting the importance of maintaining muscle function in this population [53]. In conclusion, this pilot study suggests that a supervised, online, home-based eccentric bodyweight exercise program is feasible, well tolerated, and associated with improvements in hepatic steatosis, central adiposity, and physical function in adults with MASLD. While the findings are encouraging, the small sample size and quasi-randomized design mean that the results should be interpreted as preliminary. Larger randomized controlled trials with adequate sample sizes and longer follow-up are needed to confirm the efficacy and long-term sustainability of this intervention.

## Data Availability

The data supporting the findings of this study are available from the corresponding author upon reasonable request.

## Abbreviations

ALT: alanine aminotransferase
AST: aspartate aminotransferase
BIA: bioelectrical impedance analysis
BMI: body mass index
CAP: controlled attenuation parameter
DEXA: dual-energy X-ray absorptiometry
EASL: European Association for the Study of the Liver
Fib-4: fibrosis-4 index
FLI: fatty liver index
GGT: gamma-glutamyl transferase
GLUT4: glucose transporter type 4
HbA1c: glycosylated hemoglobin
HCC: hepatocellular carcinoma
HDL: high-density lipoprotein
HDL-C: high-density lipoprotein cholesterol
HOMA-IR: homeostasis model assessment of insulin resistance
IL-6: interleukin-6
LDL: low-density lipoprotein
LDL-C: low-density lipoprotein cholesterol
LSM: liver stiffness measurement
MASH: metabolic dysfunction-associated steatohepatitis
MASLD: metabolic dysfunction-associated steatotic liver disease
NAFLD: non-alcoholic fatty liver disease
SD: standard deviation
SPSS: Statistical Package for the Social Sciences
T2DM: type 2 diabetes mellitus
TC: total cholesterol
TE: transient elastography
TG: triglyceride
TyG-WC: triglyceride glucose waist index
WC: waist circumference
